# Beyond scarring: immune-epithelial crosstalk in wound-induced hair follicle neogenesis

**DOI:** 10.3389/fimmu.2026.1872547

**Published:** 2026-07-15

**Authors:** Ying-Ming Ma, Wei Shen, Xiao-Lei Xie, Shun-Li Tang, Yuan Wu, Hua-Jie Zhong, Qiang Yan, Hui Sun

**Affiliations:** 1 Department of Dermatology, Huzhou Central Hospital, Fifth School of Clinical Medicine of Zhejiang Chinese Medical University, Huzhou, Zhejiang, China; 2 Department of Dermatology, Huzhou Central Hospital, Affiliated Central Hospital of Huzhou University, Huzhou, Zhejiang, China; 3 Huzhou Central Hospital, Fifth School of Clinical Medicine of Zhejiang Chinese Medical University, Huzhou, Zhejiang, China

**Keywords:** immune-epithelial crosstalk, precision medicine, regulatory T cells, skin microbiome, skin regeneration, wound-induced hair neogenesis (WIHN), γδ T cells

## Abstract

Wound-induced hair follicle neogenesis (WIHN) represents a remarkable regenerative phenomenon observed in adult mammalian skin (predominantly studied in mice), in which large full-thickness wounds bypass fibrotic scarring to generate fully functional *de novo* hair follicles. This process reflects the context-dependent reactivation of embryonic morphogenetic programs, driven by a coordinated tripartite immune–microbial–epithelial axis (defined here as the integrated multi-directional signaling network among localized immune cells, epithelial stem cells, and the skin microbiota/fibroblasts). Mechanistically, γδ T cells initiate dermal fibroblast reprogramming through an FGF9-Wnt feed-forward loop, while macrophages promote AKT/β-catenin signaling in Lgr5^+^ epithelial stem cells via TNF-α-driven non-canonical pathways. Regulatory T cells (Tregs) further support follicular morphogenesis by delivering Jagged1/Notch signals. In parallel, the skin microbiota acts as a key amplifier of regeneration, modulating the wound microenvironment through the IL-1β/MyD88 signaling axis. Taken together, WIHN illustrates the remarkable functional plasticity of immune signaling, which can be repurposed from host defense to orchestrating tissue regeneration. By elucidating this dynamic murine skin–immune dialogue, this mini-review provides a conceptual framework for speculative precision immunomodulatory therapies and emerging translational approaches—such as laser-assisted tissue remodeling—aimed at treating inflammatory and scarring alopecia, as well as achieving scarless and functionally restorative wound healing.

## 1 Introduction

The skin is a structurally and functionally complex organ composed of the epidermis, dermis, and specialized appendages, including hair follicles and sweat glands (1). Beyond its classical roles as a physical barrier and sensory interface, the skin operates as an active immunological organ, integrating signals from resident and circulating immune cells to maintain tissue homeostasis (2).

In most adult mammals, wound healing follows a highly conserved trajectory culminating in fibrosis and scar formation. This outcome reflects a default pro-inflammatory and pro-contractile repair program, in which rapid barrier restoration is prioritized over structural regeneration. In contrast, inflammatory skin diseases such as psoriasis and atopic dermatitis exemplify dysregulated maladaptive immune communication, which we define as persistent, skewed immune signaling that drives chronic tissue inflammation and pathological remodeling rather than healing (3, 4).

WIHN challenges this paradigm by demonstrating that adult skin retains a latent regenerative capacity. In WIHN, large wounds undergo a fate switch from fibrotic repair to organ regeneration, recapitulating key aspects of embryonic hair follicle morphogenesis (5, 6). This process reveals that wound healing outcomes are not fixed but are instead determined by the context-dependent interpretation of immune signals.

Accordingly, WIHN can be conceptualized as a model of productive immune communication, which we define as a state in which immune cells do not merely resolve injury but actively instruct surrounding epithelial and mesenchymal cells to harmoniously re-enter regenerative and morphogenetic programs (7). This raises a central question: how does the immune system transition from a pro-fibrotic defense mode to a pro-regenerative morphogenetic program? Recent advances in single-cell and spatial multi-omics technologies have begun to address this question, revealing that WIHN is governed by a coordinated tripartite interaction among immune cells, epithelial stem cells, and the microbiota (8, 9). Within this framework, immune cells function as dynamic regulators that integrate injury signals and orchestrate tissue patterning (7, 10).

## 2 The molecular choreography of skin-immune “talk”

**Figure 1:**
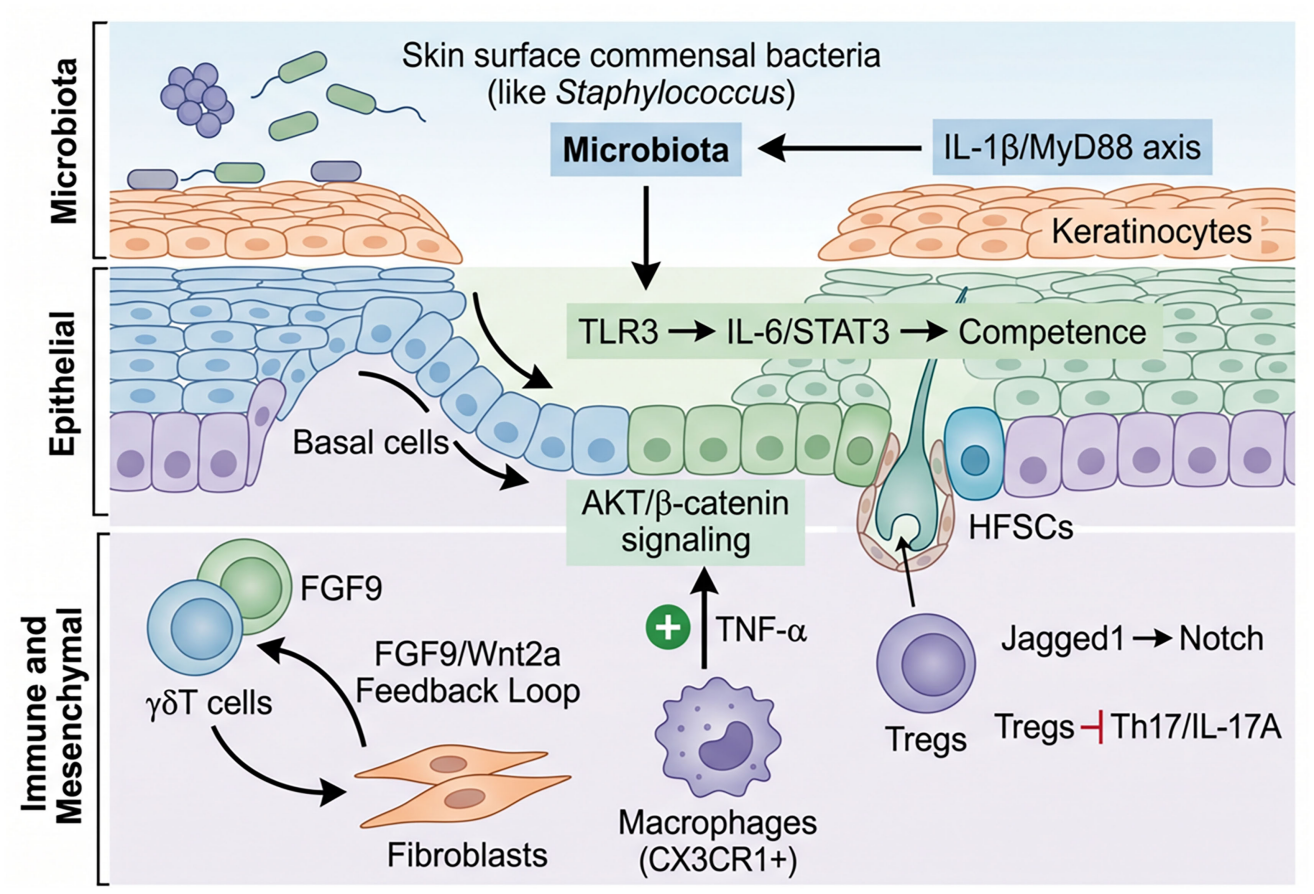
Proposed model of the tripartite immune–microbial–epithelial axis in wound-induced hair follicle neogenesis (WIHN) based on murine data. The schematic illustrates the complex multicellular dialogue required for functional skin regeneration following large full-thickness wounding. WIHN is governed by coordinated signals across three integrated compartments: The Microbial-Epithelial Interface: Commensal microbiota (e.g., *Staphylococcus*) prime the regenerative niche by activating the IL-1β/MyD88 axis in keratinocytes. In the wound center, epithelial cells sense injury-associated signals via TLR3, triggering the IL-6/STAT3 pathway to confer “regenerative competence” to the wound epidermis. Dermal Reprogramming: Resident γδ T cells serve as early initiators by secreting FGF9, which acts on dermal fibroblasts to establish an FGF9/Wnt2a feedback loop. This crosstalk reprograms fibroblasts into an inductive, dermal papilla-like state essential for follicle initiation. Macrophage-Mediated Morphogenesis: CX3CR1+ macrophages accumulate in the wound bed and release TNF-α, which non-canonically activates AKT/β-catenin signaling in Lgr5+ hair follicle stem cells (HFSCs) at the wound edge, driving placode formation. Treg-Mediated Microenvironmental Modulation: Regulatory T cells (Tregs) are hypothesized to support follicular morphogenesis via Jagged1-mediated Notch activation in hair follicle stem cells (HFSCs), while concurrently attenuating the Th17/IL-17A pathway to mitigate excessive fibrotic scarring.

Recent advances in single-cell transcriptomics, spatial transcriptomics, and multi-omics technologies have revolutionized our understanding of WIHN (10, 11). These studies demonstrate that WIHN is not merely a classic epithelial–mesenchymal interaction (EMI), but rather a highly orchestrated tripartite dialogue among immune cells, epithelial stem cells, and the skin microbiota (12–14). In this dynamic “skin–immune talk,” immune cells function as central coordinators that sense injury, integrate environmental signals, and redirect wound fibroblasts and epithelial progenitors from a fibrotic default program toward embryonic-like organ regeneration (15, 16). This molecular choreography converts inflammatory cues into precise morphogenetic instructions, determining whether the wound heals with scarring or restores functional skin appendages (**Figure 1**) (17).

### 2.1 Resident T cells and fibroblast reprogramming

Resident T cells, particularly the dermal γδ T cell subset, act as key cellular initiators of the regenerative response in murine models. Following cutaneous injury, these dermal γδ T cells rapidly secrete fibroblast growth factor 9 (FGF9), which stimulates adjacent dermal fibroblasts to activate a Wnt2a-dependent positive feedback loop (7, 11, 18, 19). This intercellular crosstalk reprograms default wound-bed fibroblasts into regeneration-competent, dermal papilla-like cells that are crucial for initiating *de novo* hair follicle formation in mice. While epidermal dendritic epidermal T cells (DETCs) also participate in early wound surveillance and barrier re-establishment, their broader immunomodulatory signaling is distinct from this localized, dermal γδ T cell-mediated FGF9 cascade (20).

In the absence of this γδ T cell-derived paracrine signal, the surrounding dermal niche fails to acquire the inductive properties required for hair placode initiation. Consequently, resident dermal γδ T cells bridge the early inflammatory response and the subsequent morphogenetic phase, effectively translating acute injury cues into spatial and molecular instructions that reactivate embryonic developmental programs in adult murine skin (10, 20).

### 2.2 Macrophages: from debridement to morphogenesis

Macrophages, traditionally viewed primarily as phagocytic cells responsible for debris clearance, play a far more sophisticated role in the WIHN microenvironment (21). A specific subset of CX3CR1^+^ macrophages accumulate in the regenerative wound bed and adopts a non-canonical secretory phenotype (22). These cells release TNF-α, which paradoxically does not trigger apoptosis or sustained inflammation. Instead, TNF-α activates the AKT/β-catenin signaling pathway in Lgr5^+^ epithelial stem cells residing in the wound edge (23).

This macrophage–epithelial crosstalk is indispensable for hair follicle placode formation and subsequent downgrowth. It exemplifies a key principle of regenerative biology: the same cytokine can exert diametrically opposite effects depending on the cellular context and timing. In the WIHN setting, inflammatory signals are “reinterpreted” as morphogenetic cues, converting a potentially destructive response into one that drives organized tissue patterning (6, 10, 24).

### 2.3 Tregs and the maintenance of the regenerative window

Regulatory T cells (Tregs) are preferentially recruited to and retained within the hair follicle-rich regenerative niche, where they are hypothesized to foster a permissive microenvironment for tissue morphogenesis. Specifically, evidence from murine models indicates that Tregs can modulate hair follicle stem cell (HFSC) dynamics during repair, potentially facilitating their activation and lineage commitment via Jagged1/Notch signaling (25). In addition to these direct epithelial interactions, Tregs serve as crucial immunoregulatory modulators that restrain hyper-inflammatory and pro-fibrotic signaling cascades, with a notable suppressive effect on the Th17/IL-17A axis (26). By restraining neutrophil over-recruitment and limiting TGF-β-driven collagen cross-linking, Tregs help maintain a transient regenerative window—a specific, limited temporal phase post-wounding when the tissue microenvironment remains receptive to morphogenetic cues before the onset of dominant fibrotic scarring (27, 28).

In the absence of sufficient Treg activity, the wound microenvironment shifts rapidly toward scarring, underscoring the delicate balance Tregs maintain between immune tolerance and regenerative instruction (29, 30). Their presence thus ensures that the early regenerative signals initiated by γδ T cells and macrophages can be sustained long enough for complete *de novo* hair follicle development (11, 31, 32).

### 2.4 Skin microbiome: an external layer of regulation

The skin microbiota constitutes a critical external layer within the regenerative interactome of WIHN. Far beyond acting as passive colonizers, commensal microbes actively participate in the tripartite immune–microbial–epithelial axis by translating environmental cues into molecular signals that modulate regeneration (8, 9).

Upon skin injury, commensal bacteria such as *Staphylococcus* species engage pattern recognition receptors on keratinocytes, triggering the production and release of interleukin-1β (IL-1β). This cytokine then activates the MyD88-dependent signaling pathway in keratinocytes, effectively priming both the innate immune response and the epithelial stem cell compartment. The IL-1β/MyD88 axis enhances the sensitivity of the wound bed to subsequent regenerative signals, creating a permissive microenvironment for hair follicle neogenesis (8, 12, 13, 33).

Compelling evidence from germ-free mouse models underscores the indispensable role of the microbiota. Animals raised in the absence of commensal microbes exhibit significantly impaired WIHN, with reduced formation of *de novo* hair follicles despite comparable wound closure rates. Restoration of a conventional microbiota or topical application of specific commensals (including both commensal and select pathogenic strains such as *Staphylococcus aureus*) can rescue regenerative capacity, demonstrating that microbial signals are both necessary and sufficient to promote robust neogenesis (34).

Thus, the skin microbiome functions as a dynamic environmental sensor and amplifier. It integrates exogenous stimuli—such as barrier breach and microbial density—into the skin–immune dialogue, fine-tuning the balance between inflammation and morphogenesis. By modulating the IL-1β/MyD88 axis, commensals help ensure that early wound signals favor regeneration over fibrosis, highlighting the microbiota as an essential, yet often overlooked, regulator of adult mammalian organ regeneration.

## 3 Translational potential: beyond the rodent model

While much of our mechanistic understanding of WIHN derives from rodent models, emerging evidence supports its translational relevance to human skin (6, 10). The recent paradigm shifts in treating inflammatory skin diseases—from broad systemic immunosuppression to precision biologics targeting specific pathways (e.g., TNF, IL-17, and IL-23 inhibitors)—provides a valuable blueprint for developing WIHN-inspired regenerative therapies (11, 12, 35).

### 3.1 Precision immunotherapy

By harnessing key molecular nodes identified in the WIHN axis, researchers aim to investigate whether it is possible to pharmacologically reprogram adult skin from a fibrotic to a regenerative state. Small-molecule agonists of TLR3 or FGF9 signaling, as well as bioengineered Notch ligands (e.g., Jagged1 mimetics), represent promising translational ideas that could hypothetically activate the immune–epithelial regenerative circuitry in chronic wounds or alopecia-affected scalp tissue. These approaches are designed to determine whether the “productive immune communication” characteristic of WIHN can be recapitulated in human skin, with the long-term objective of stimulating *de novo* hair follicle formation or reviving dormant follicles in conditions such as androgenetic alopecia and scarring alopecia (11, 28, 30).

### 3.2 Laser and device-based modulation

Fractional laser resurfacing and other energy-based devices offer an interesting clinical tool, though their ability to induce true *de novo* hair follicle neogenesis in humans remains unproven. In clinical settings, these devices are used to promote tissue remodeling and may stimulate surviving dormant follicles. However, they have not been shown to reliably trigger the complete embryogenesis observed in mouse WIHN. When combined with adjunctive therapies—such as microbiome-derived postbiotics, topical IL-1β modulators, or Treg-enhancing agents—these approaches are being explored as speculative future strategies to optimize the wound environment. This combinatorial framework represents a potential future direction, rather than an evidence-based clinical treatment, for refractory inflammatory and scarring alopecia, including Alopecia Areata and Cicatricial Alopecia (e.g., lichen planopilaris and frontal fibrosing alopecia). By converting the wound microenvironment from pro-fibrotic to pro-regenerative, such strategies may enable scarless healing and functional restoration of hair follicles, representing a transformative advance beyond current symptomatic treatments (9, 36–38).

Ultimately, translating WIHN biology into clinical applications presents substantial hurdles due to profound species-specific differences. The primary translational barriers encompass discrepancies in immune architecture (e.g., the relative scarcity of epidermal γδ T cells in humans compared to mice), distinct wound microenvironments, and the intrinsic pro-fibrotic and contractile healing trajectory of human skin, which prioritizes rapid barrier restoration and scarring over the regeneration of functional appendages. Bridging the gap between rodent findings and human physiology will necessitate the utilization of advanced organoid platforms, humanized mouse models, and meticulously designed clinical trials (15, 39).

## 4 Discussion

**Figure 2:**
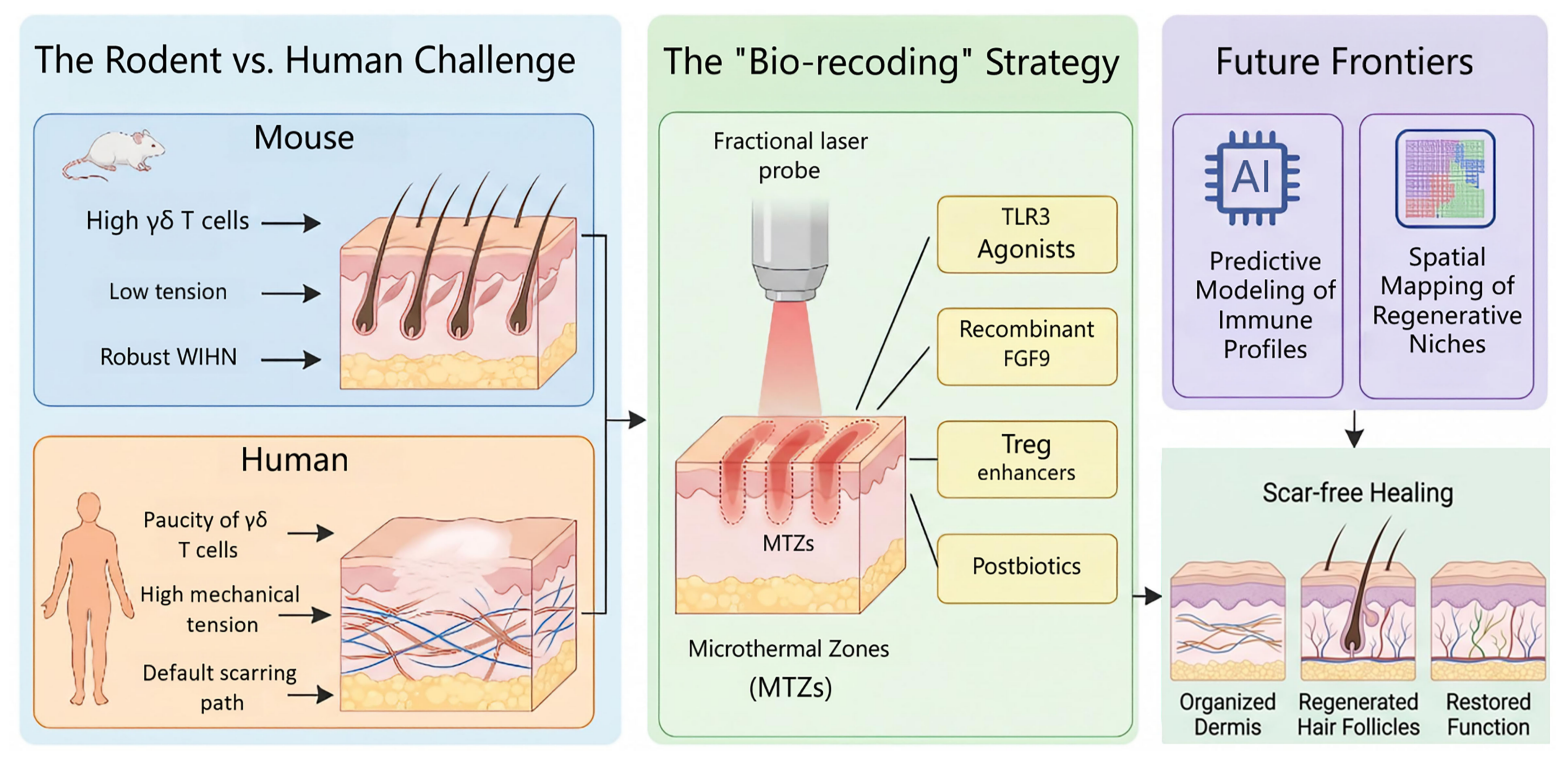
Bridging the gap in regenerative dermatology: species-specific insights and translational frameworks. The schematic illustrates the challenges and emerging strategies for translating wound-induced hair follicle neogenesis (WIHN) from bench to bedside. The Rodent vs. Human Challenge: A fundamental disparity exists between species. Mouse skin (top left) facilitates robust WIHN due to a high density of resident γδ T cells and low mechanical tension. In contrast, human skin (bottom left) possesses a paucity of γδ T and experiences high mechanical tension, which steers the wound healing process toward a default fibrotic scarring path characterized by dense collagen deposition and loss of appendages. The “Bio-recoding” Strategy: To bypass these evolutionary constraints, a combinatorial approach is proposed. Fractional laser probes generate controlled micro-injuries known as microthermal zones (MTZs), which mimic the mechanical and molecular triggers of WIHN. This stimulus can be augmented by a “bio-recoding” cocktail consisting of TLR3 agonists (to simulate damage sensing), recombinant FGF9 (to compensate for γδ T cell scarcity), Treg enhancers (to maintain the regenerative window), and microbiome-derived postbiotics (to prime the immune-epithelial axis). Future Frontiers: Emerging technologies are essential for personalizing these therapies. AI-driven predictive modeling of patient-specific immune profiles and spatial mapping of regenerative niches enable the precise orchestration of the skin–immune dialogue. Scar-free Healing (Ultimate Therapeutic Objective): The long-term conceptual goal of transitioning human wound repair from symptomatic scarring to complete, functional tissue restoration, characterized by an organized dermis, regenerated hair follicles, and fully restored cutaneous functions (such as innervation and sweat gland activity).

The discovery of WIHN fundamentally challenges the long-standing dogma that adult mammalian skin wounds must inevitably heal by fibrosis and scarring. By demonstrating that large wounds can reactivate embryonic morphogenetic programs through a coordinated tripartite immune–microbial–epithelial dialogue, WIHN reveals that regeneration is not lost in adulthood but can be conditionally unlocked. Nevertheless, significant biological and technical barriers must be overcome before this regenerative “conversation” can be reliably translated from rodent models into effective human therapies (**Figure 2**) (27, 40).

### 4.1 Current research gaps

The most critical obstacle to clinical translation lies in the substantial immunological and anatomical differences between rodent and human skin (11, 41).

In murine models, WIHN is highly dependent on resident γδ T cells—specifically dermal γδ T cells, which serve as the primary source of regenerative FGF9. Conversely, human skin harbors a significantly lower density of γδ T cells; rather than being distributed throughout the epidermis, these cells are predominantly localized within vascularized dermal compartments. Consequently, human cutaneous immunity relies more heavily on αβ T cells and the Vδ1^+^ γδ subset, establishing a fundamentally distinct injury-sensing and signal-amplification network (42).

Furthermore, the “regenerative window” in humans is considerably narrower due to a stronger default pro-fibrotic and high-tension mechanical environment (12, 14). Unlike the African spiny mouse (Acomys), which forms blastema-like aggregates of lineage-restricted progenitors to achieve nearly scar-free healing, human wounds rapidly progress to myofibroblast-dominated contraction and hypertrophic scarring (43, 44).

Key mechanistic questions remain unresolved: how can we artificially lower the human inflammatory threshold to allow regenerative signals—such as non-canonical TNF-α, AKT/β-catenin, and IL-6/STAT3 pathways—to predominate over classical pro-fibrotic cascades (e.g., TGF-β/Smad and IL-17-driven collagen cross-linking) (6, 40)? Without a deeper understanding of these species-specific differences, strategies optimized in mice may prove insufficient or even counterproductive in humans (15).

### 4.2 Translational implications

Despite these challenges, the immune-epithelial axis elucidated in WIHN provides a compelling roadmap for treating refractory alopecia and chronic non-healing wounds (45). Rather than relying on broad immunosuppression, future therapies can focus on reprogramming the wound microenvironment toward productive regeneration (11).

#### 4.2.1 Targeted biologics and small molecules

Next-generation approaches may include selective Wnt pathway activators, TLR3 agonists to mimic early damage sensing, FGF9 analogs, and Jagged1/Notch pathway biologics. These agents could directly stimulate follicle stem cells and dermal papilla reconstitution while bypassing the scarcity of resident γδ T cells. Such precision immunomodulation represents a paradigm shift from current corticosteroid or JAK-inhibitor therapies for Alopecia Areata toward therapies that actively instruct regeneration (11, 13, 15, 46, 47).

#### 4.2.2 Synergistic device-based therapy

Energy-based and micro-injury devices, such as fractional lasers and microneedling, have demonstrated clinical utility in stimulating hair regrowth by generating controlled micro-wounds. However, while these modalities successfully initiate local tissue remodeling and enhance the penetration of topical agents, they have not yet been shown to trigger true *de novo* organogenesis in humans. Consequently, integrating these physical interventions with biological adjuvants—such as recombinant FGF9, adipose-derived stem cell secretomes, microbiome-derived postbiotics, or Treg-modulating agents—presents a promising, albeit speculative, multimodal framework. Such combination strategies merit future investigation to determine whether the human wound microenvironment can be modulated to support compromised follicles and attenuate fibrotic scarring (10, 38, 48–50).

### 4.3 Future developments

The future of WIHN research will be driven by the integration of advanced technologies capable of decoding the spatiotemporal complexity of the skin–immune interactome.

#### 4.3.1 Spatial transcriptomics and cellome mapping

High-resolution spatial transcriptomics and multiplexed imaging will enable researchers to map “multicellular neighborhoods” in real time, identifying the precise locations, timing, and cellular partners involved in the regenerative dialogue (31, 51–53). Such mapping will reveal hidden regenerative niches and allow the discovery of novel biomarkers that distinguish regenerative versus fibrotic trajectories (54).

#### 4.3.2 AI and machine learning

Artificial intelligence (AI) will play a pivotal role in integrating multi-omics datasets, including the gut–immune–skin axis (55–57). Predictive models trained on these large-scale datasets can guide the design of personalized “regenerative cocktails” tailored to individual patients’ immune profiles, microbiome composition, and genetic background (55, 58). This precision medicine approach will optimize the balance between suppressing pathological inflammation and promoting localized morphogenesis (59–61). In summary, WIHN not only illuminates fundamental principles of adult tissue regeneration but also offers a transformative framework for regenerative dermatology. By systematically addressing species-specific barriers and leveraging cutting-edge technologies, the transition from fibrotic repair to functional regeneration in human skin may finally become clinically achievable.

## 5 Conclusion

In summary, evidence from murine models of WIHN demonstrates that the intricate crosstalk within the immune–epithelial–microbial axis functions as a master regulatory node governing the balance between default fibrotic repair and true tissue regeneration. By deciphering this dynamic cellular dialogue, researchers have begun to identify novel pathways that could theoretically be targeted to promote scarless healing and functional follicle restoration. Harnessing these regenerative signals holds promising translational potential for exploring novel therapeutic strategies in alopecia and chronic scarring disorders.

However, translating these rodent-derived mechanisms to human clinical applications remains a substantial challenge. Pronounced species-specific barriers—most notably discrepancies in skin-resident immune cell populations, differing wound microenvironments, and the highly contractile, pro-fibrotic healing trajectory of human skin—must be systematically addressed before clinical therapies can be safely realized (15, 39).

Looking forward, the prospective convergence of precision immunomodulation, energy-based devices, microbiome engineering, and spatial multi-omics technologies offers a compelling framework to investigate next-generation regenerative therapies. Rather than viewing scarring as an inevitable default of adult human tissue repair, these emerging, multidisciplinary strategies seek to explore how local environments can be modulated to support functional tissue reconstruction. As future research continues to systematically bridge the gap between rodent findings and human physiology, the long-standing biological boundary between simple wound repair and true organ regeneration may finally be explored.

## Glossary

AI: Artificial Intelligence
CX3CR1⁺: CX3C chemokine receptor 1 positive
DETCs: Dendritic Epidermal T Cells
EMI: Epithelial–Mesenchymal Interaction
FGF9: Fibroblast Growth Factor 9
HFSC: Hair Follicle Stem Cell
IL-1β: Interleukin-1β
IL-17A: Interleukin-17A
IL-23: Interleukin-23
JAK: Janus Kinase
Lgr5: Leucine-rich repeat-containing G-protein coupled receptor 5
MyD88: Myeloid differentiation primary response 88
STAT3: Signal Transducer and Activator of Transcription 3
TGF-β: Transforming Growth Factor Beta
Th17: T helper 17 cells
Th2: T helper 2 cells
TLR3: Toll-Like Receptor 3
TNF-α: Tumor Necrosis Factor Alpha
Tregs: Regulatory T cells
WIHN: Wound-Induced Hair Follicle Neogenesis
T cells: Gamma-delta T cells
T cells: Alpha-beta T cells
Vδ1⁺: V delta 1 positive subset
